# Testing Internal Quality Control of Clinical Laboratory Data Using Paired *t*-Test under Uncertainty

**DOI:** 10.1155/2021/5527845

**Published:** 2021-09-04

**Authors:** Mohammed Albassam, Muhammad Aslam

**Affiliations:** Department of Statistics, Faculty of Science, King Abdulaziz University, Jeddah 21551, Saudi Arabia

## Abstract

The existing paired *t*-test under classical statistics cannot be applied when the data is obtained from the complex process, having interval, uncertainty, indeterminacy, and incompleteness. In this paper, the modification of the paired *t*-test under neutrosophic statistics is proposed. The testing criterion of the proposed paired *t*-test is given. The application of the proposed paired *t*-test is given using the interval quality control of clinical laboratory data. From the analysis, it can be seen than the proposed test is quite effective and informative to apply for testing the measurement tools in the clinical laboratory.

## 1. Introduction

The statistical tests including *t*-test and *z*-test have been widely applied in a variety of fields where the sampling is done and the experimenters are interested in testing the population parameter on the basis of sample information. According to Feng et al. [1], “T-test, which is also called the Student's t-test is often used as a statistical method to assess whether the mean value of the data from an independent sample which follows a normal distribution is consistent with or departs significantly from the mean value of the null hypothesis, or whether the difference between the means of two independent samples which follow a normal distribution are statistically significant” (see Rivas-Ruiz, Pérez-Rodríguez, and Talavera [2]). The paired *t*-test is applied when some sample information from the same objects is obtained twice and the sample size is less than 30 (see Hashimoto [3]). This test is applied to test whether the mean difference between paired data is zero or not. During the experimentation, it is noted that all results of the interval quality control (IQC) follow the normal distribution, with an unknown standard deviation and sample size being less than 30. Therefore, the paired *t*-test can be applied for testing the IQC of clinical laboratory data measured from the two instruments (Westgard and Hunt [4] and Ceriotti, Brugnoni, and Mattioli [5]). More applications about the statistical tests can be seen in Westgard and Hunt [4], Maghsoodloo and Huang [6], and Niwitpong and Niwitpong [7].

The *t*-test and paired *t*-test under classical statistics are designed under the assumption that sample data having determined, precise, exact, and certain observations should be selected from the normal distribution. Viertl [8] stated that “statistical data are frequently not precise numbers but more or less non-precise, also called fuzzy. Measurements of continuous variables are always fuzzy to a certain degree.” When the data is in intervals, uncertain, and vague, the existing tests under classical statistics can be applied for testing the hypothesis about the population means. In such a situation, the tests under fuzzy logic are needed. The idea of a *p* value under fuzzy logic was discussed by Filzmoser and Viertl [9]. The paired *t*-test using the fuzzy logic can be seen in Tsai and Chen [10]. Taheri and Arefi [11] proposed the fuzzy-based test for testing the mean of the population. The details about the fuzzy-based tests can be seen in Jamkhaneh and Ghara [12], Chachi, Taheri, and Viertl [13], Kalpanapriya and Pandian [14], Montenegro et al. [15], and Park, Lee, and Jun [16].

The fuzzy logic only deals with the measure of truth and the measure of falseness. This logic ignores the measure of indeterminacy that is important in an uncertain environment. To overcome this issue, Smarandache [17] introduced the neutrosophic logic which gives the information about three measures including truth, falseness, and indeterminacy. Smarandache [18] also proved the efficiency of the neutrosophic logic over the fuzzy logic and interval analysis. Broumi and Smarandache [19], Guo and Sengur [20], Broumi, Bakali, Talea, and Smarandache [21], Abdel-Baset, Chang, and Gamal [22], and Abdel-Basset, Mohamed, Elhoseny, Chiclana, and Zaied [23] can be referred for further investigation about the neutrosophic logic. The neutrosophic statistics using the idea of neutrosophic logic was introduced by Smarandache [24]. Chen, Ye, and Du [25] and Chen, Ye, Du, and Yong [26] introduced the idea of neutrosophic form to deal with the neutrosophic numbers. Aslam [27], Aslam and Albassam [28], and Aslam [29] proposed statistical tests under neutrosophic statistics.

The paired *t*-test under classical statistics cannot be applied for testing the difference of means when the data have neutrosophic numbers. On the other hand, the tests based on fuzzy logic do not give information about the measure of indeterminacy. Therefore, there is need to develop the paired *t*-test under neutrosophic statistics to deal with the testing of population means. In this paper, the design of the paired *t*-test is introduced first. The operational procedure of the proposed paired *t*-test will be given. The application of the proposed test will be given using the IQC of the clinical laboratory. It is expected that the proposed paired test will be efficient than the existing paired test under classical in terms of a measure of indeterminacy, information, and flexibility.

## 2. Preliminaries

Let *X*_1*N*_ = *X*_1*L*_ + *X*_1*U*_*I*_1*N*_; *I*_1*N*_*ϵ*[*I*_1*L*_, *I*_1*U*_] and *X*_2*N*_ = *X*_2*L*_ + *X*_2*U*_*I*_2*N*_; *I*_2*N*_*ϵ*[*I*_2*L*_, *I*_2*U*_] be a set of paired neutrosophic observations of size *n*_*N*_*ϵ*[*n*_*L*_, *n*_*U*_], where *X*_1*L*_, *X*_2*L*_ denote the determined observations; *X*_1*U*_*I*_1*N*_, *X*_2*U*_*I*_2*N*_ are indeterminate observations; and *I*_1*N*_*ϵ*[*I*_1*L*_, *I*_1*U*_], *I*_2*N*_*ϵ*[*I*_2*L*_, *I*_2*U*_] show the indeterminacy intervals associated with neutrosophic random variables *X*_1*N*_*ϵ*[*X*_1*L*_, *X*_1*U*_] and *X*_2*N*_*ϵ*[*X*_2*L*_, *X*_2*U*_], respectively. Suppose that *D*_*N*_*ϵ*[*D*_*L*_, *D*_*U*_] is a neutrosophic variable that shows the difference between *X*_1*N*_*ϵ*[*X*_1*L*_, *X*_1*U*_] and *X*_2*N*_*ϵ*[*X*_2*L*_, *X*_2*U*_]. Based on the information, the neutrosophic average of the neutrosophic variable *D*_*N*_*ϵ*[*D*_*L*_, *D*_*U*_] is defined as follows:

(1) $$\begin{array}{c} {\bar{D}}_{N}={\bar{D}}_{L}+{\bar{D}}_{N}I_{N};I_{\bar{D}N}\epsilon \left[I_{\bar{D}L},I_{\bar{D}U}\right], \end{array}$$

where ${\bar{D}}_{N}=\sum D_{N}/n_{N};{\bar{D}}_{N}\epsilon \left[{\bar{D}}_{L},{\bar{D}}_{U}\right]\;$and $I_{\bar{D}N}\epsilon \left[I_{\bar{D}L},I_{\bar{D}U}\right]$ are an indeterminate interval associated with *D*_*N*_*ϵ*[*D*_*L*_, *D*_*U*_].

The sum of the square of deviation of *D*_*N*_*ϵ*[*D*_*L*_, *D*_*U*_] from ${\bar{D}}_{N}\epsilon \left[{\bar{D}}_{L},{\bar{D}}_{U}\right]$ is given by

(2) $$\begin{array}{c} \overset{n_{N}}{\underset{i=1}{\sum }}{\left(D_{i}-{\bar{D}}_{\text{iN}}\right)}^{2}=\left[\begin{array}{c} \min\left(\begin{array}{c} \left(D_{Li}+D_{Ui}I_{L}\right)\left({\bar{D}}_{L}+{\bar{D}}_{U}I_{L}\right),\left(D_{Li}+D_{Ui}I_{L}\right)\left({\bar{D}}_{L}+{\bar{D}}_{U}I_{U}\right)\\ \left(D_{Li}+D_{Ui}I_{U}\right)\left({\bar{D}}_{L}+{\bar{D}}_{U}I_{L}\right),\left(D_{Li}+D_{Ui}I_{U}\right)\left({\bar{D}}_{L}+{\bar{D}}_{U}I_{U}\right) \end{array}\right)\\ \max\left(\begin{array}{c} \left(D_{Li}+D_{Ui}I_{L}\right)\left({\bar{D}}_{L}+{\bar{D}}_{U}I_{L}\right),\left(D_{Li}+D_{Ui}I_{L}\right)\left({\bar{D}}_{L}+{\bar{D}}_{U}I_{U}\right)\\ \left(D_{Li}+D_{Ui}I_{U}\right)\left({\bar{D}}_{L}+{\bar{D}}_{U}I_{L}\right),\left(D_{Li}+D_{Ui}I_{U}\right)\left({\bar{D}}_{L}+{\bar{D}}_{U}I_{U}\right) \end{array}\right) \end{array}\right],\;I_{N}\epsilon \left[I_{L},I_{U}\right]. \end{array}$$

The neutrosophic variance, say *S*_*DN*_^2^*ϵ*[*S*_*DL*_^2^, *S*_*DU*_^2^], is defined by

(3) $$\begin{array}{c} S_{DN}^{2}=\frac{\sum_{i=1}^{n_{N}}{\left(D_{i}-{\bar{D}}_{\text{iN}}\right)}^{2}}{n_{N}-1},\;n_{N}\epsilon \left[n_{L},n_{U}\right],{\bar{D}}_{N}\epsilon \left[{\bar{D}}_{L},{\bar{D}}_{U}\right]. \end{array}$$

The neutrosophic standard deviation *S*_*DN*_*ϵ*[*S*_*DL*_, *S*_*DU*_] is defined by

(4) $$\begin{array}{c} S_{DN}=\sqrt{\frac{\sum_{i=1}^{n_{N}}{\left(D_{i}-{\bar{D}}_{\text{iN}}\right)}^{2}}{n_{N}-1}\;},\;n_{N}\epsilon \left[n_{L},n_{U}\right],{\bar{D}}_{N}\epsilon \left[{\bar{D}}_{L},{\bar{D}}_{U}\right]. \end{array}$$

## 3. The Proposed Paired *t*-Test

As mentioned before, the paired *t*-test is applied when the related observations from the same individual are recorded under the assumption of randomness. In such a case, the paired *t*-test is applied to see either that the difference between paired observations is zero or not. The existing paired *t*-test under classical statistics is applied to see the difference when all observations in paired data are determined, well-defined, and certain and exact. The existing paired *t*-test cannot be applied when the paired data information is recorded in indeterminacy intervals. In this section, the modified form of the existing paired *t*-test under classical statistics is presented under the neutrosophic statistics. The proposed paired *t*-test will work under the assumption that paired data is recorded in a random way and observations have indeterminate intervals. The main aim to propose the paired *t*-test under neutrosophic statistics to see that the mean difference between the paired data is significantly different from zero. Suppose that *μ*_*DN*_ denotes the difference between the means of paired data. The neutrosophic null hypothesis *H*_0*N*_ and neutrosophic alternative hypothesis are stated as follows:

(5) $$\begin{array}{c} \left\{\begin{array}{c} H_{0N}:\mu_{DN}=D_{0N},\\ H_{1N}:\mu_{DN}\neq D_{0N}. \end{array}\right. \end{array}$$

The test statistic for the proposed paired *t*-test is given by

(6) $$\begin{array}{c} t_{N}=\frac{{\bar{D}}_{N}-D_{0N}}{S_{DN}/\sqrt{n_{N}}},\;{\bar{D}}_{N}\epsilon \left[{\bar{D}}_{L},{\bar{D}}_{U}\right],S_{DN}\epsilon \left[S_{DL},S_{DU}\right],n_{N}\epsilon \left[n_{L},n_{U}\right]. \end{array}$$

The neutrosophic form of the test statistic *t*_*N*_*ϵ*[*t*_*L*_, *t*_*U*_] of the proposed test can be expressed as follows:

(7) $$\begin{array}{c} t_{N}=t_{L}+t_{U}I_{tN},\;I_{tN}\epsilon \left[I_{\text{t}L},I_{tU}\right], \end{array}$$

where *t*_*L*_ shows the test statistic of the paired *t*-test under classical statistics. This part of a neutrosophic form is known as the determined part, and *t*_*U*_*I*_*tN*_; *I*_*tN*_*ϵ*[*I*_*tL*_, *I*_*tU*_] denotes the indeterminate part associated with the measure of indeterminacy *I*_*tN*_*ϵ*[*I*_*tL*_, *I*_*tU*_]. Note here that the proposed test statistic reduces the statistic under classical statistics when  *I*_*tL*_ = 0. The statistical test can be carried out using the following steps.

**Step 1 .**

State *H*_0*N*_ and *H*_1*N*_.

**Step 2 .**

State level of significance *α*.

**Step 3 .**

Compute the values of the test statistic *t*_*N*_*ϵ*[*t*_*L*_, *t*_*U*_].

**Step 4 .**

Select critical value at *α* and degree of freedom *γ*_*N*_ = *n*_*N*_ − 1 from Kanji [30].

**Step 5 .**

Do not reject *H*_0*N*_ if the calculated value of  *t*_*N*_*ϵ*[*t*_*L*_, *t*_*U*_] is less than the critical value is obtained in Step 4.

## 4. Real Example from Clinical Laboratory

The application of the proposed paired *t*-test will be given using the internal quality control (IQC) of clinical laboratory data. The data of IQC of two instruments are selected from Feng et al. [1]. According to Feng et al. [1], “The results of IQC about luteotropichormone (LH) were collected from July 1, 2015 to July 31, 2015 in the Clinical Laboratory Center of Tumor Hospital Affiliated to Xinjiang Medicine University. LH in the laboratory was tested on two same-type of instruments (Roche Cobas e602 electrochemistry luminescence immunity analyzer, Switzerland), and the IQC of LH on the two instruments was measured by the same operator. The IQC substance was divided into two duplicates which were tested on the two instruments respectively every time. The IQC substance was LiquichekTM Immunoassay Premium Quality Control product of Bio-Rad Laboratories, Inc. (USA), and the lot numbers of IQC substance were 40,303 (high level) and 40,302 (middle level), respectively.” The data is shown in Table 1. From Table 1, it can be noted that the IQC data has lower values and higher values for middle and high levels. For the data given in the intervals, the use of the existing paired *t*-test under classical statistics is inappropriate. The proposed paired *t*-test can be applied as an alternative to the existing test. The values of *D*_*N*_*ϵ*[*D*_*L*_, *D*_*U*_] are shown in Table 2. The necessary calculations to carry out the proposed paired test are also shown in Table 2. The values *S*_*DN*_^2^*ϵ*[*S*_*DL*_^2^, *S*_*DU*_^2^] are given as

(8) $$\begin{array}{c} S_{DN}^{2}=\frac{\sum_{i=1}^{n_{N}}{\left(D_{i}-{\bar{D}}_{\text{iN}}\right)}^{2}=\left[17.37,125.99\right]}{35}=\left[0.4964,3.5998\right]. \end{array}$$

The values of *S*_*DN*_*ϵ*[*S*_*DL*_, *S*_*DU*_] are calculated as

(9) $$\begin{array}{c} S_{DN}=\sqrt{\frac{\left[17.37,125.99\right]}{35}\;}=\left[0.7045,1.8973\right]. \end{array}$$

The values of a statistic *t*_*N*_*ϵ*[*t*_*L*_, *t*_*U*_] can be computed as follows:

(10) $$\begin{array}{c} t_{N}=\frac{{\bar{D}}_{N}-D_{0N}}{S_{DN}/\sqrt{n_{N}}}=\frac{\left[-0.165,-0.6794\right]-\left[0,0\right]}{\left[0.7045,1.8973\right]/\sqrt{36}}=\left[-1.40,-2.14\right]. \end{array}$$

The proposed test is preceded as follows:

**Step 6 .**

State *H*_0*N*_ : *D*_0*N*_ = 0 and *H*_1*N*_ : *D*_0*N*_ ≠ 0.

**Step 7 .**

State level of significance *α* = 5%.

**Step 8 .**

The computed the value of the test statistic is | *t*_*N*_|*ϵ*[1.40,2.14].

**Step 9 .**

The selected critical value is 2.03 at *α* = 5% and degree of freedom *γ*_*N*_ = 34.

**Step 10 .**

Do not reject *H*_0*N*_ if the calculated value of | *t*_*N*_|*ϵ*[1.40,2.14] is less than the critical value 2.03.

From the study, it can be noted that the determined part of the data shows that the difference is 0 while the indeterminate part shows that the difference in means is not 0.

## 5. Comparative Study

In this section, the performance of the proposed paired *t*-test will be compared to the existing paired *t*-test under classical statistics in terms of the measure of indeterminacy. As mentioned before, the proposed paired *t*-test is the generalization of the existing paired *t*-test and reduces to the existing test if no uncertainty is recorded. The test statistic of the proposed paired *t*-test in the neutrosophic form can be expressed as | *t*_*N*_| = 1.40 + 2.14*I*_*tN*_; *I*_*tN*_*ϵ*[0,0.3457]. The first value  *t*_*L*_=1.40 denotes the test statistic value of the existing paired *t*-test and is obtained when *I*_*tL*_ = 0. The second part denoted the indeterminate part of the neutrosophic test statistic. From this study, the measure of indeterminacy associated with the test statistic is 0.3457. Based on this information, the proposed paired *t*-test can be interpreted as follows: the probability of rejecting*H*_0*N*_being true is 0.05, the probability of accepting *H*_0*N*_ is 0.95, and the probability of uncertainty about *H*_0*N*_ is 0.3457. From this study, it can be noted that the proposed paired *t*-test gives information about the chance of indeterminacy. In addition, the proposed test provides the results in indeterminate intervals rather than the exact value. Therefore, the proposed test is flexible and informative than the existing paired *t*-test under classical statistics.

## 6. Concluding Remarks

In this paper, the modification of the paired *t*-test under neutrosophic statistics was proposed. The testing criterion of the proposed paired *t*-test was given. The proposed test was the modification of a paired *t*-test under classical statistics. The proposed paired *t*-test is helpful to test the hypothesis of mean difference when the data is obtained from the same objects under a complex or uncertain environment. The application of the proposed paired *t*-test was given using the IQC data of the clinical laboratory. The comparative study shows that the proposed test gives information about the measure of indeterminacy which the existing paired *t*-test does not provide. In addition, the proposed paired *t*-test gives the values of the test statistic in intervals which are required under an indeterminate environment. The proposed test can be applied in business, environmental study, industry, and medical science. The proposed paired *t*-test for big data can be studied as future research. The proposed test power for various statistical distributions can be studied as future research. The extension of the proposed paired *t*-test using various sampling schemes can be considered as future research.

## Figures and Tables

**Table 1:** The IQC of clinical laboratory data.

Number of testing	No. 1 instrument (middle level) (mIU/mL)	No. 2 instrument (middle level) (mIU/mL)	No. 1 instrument (high level) (mIU/mL)	No. 2 instrument (high level) (mIU/mL)
1	18.36	19.12	61.63	62.64
2	18.77	19.07	63.11	64.36
3	18.98	19.58	66.88	66.06
4	17.97	19.35	62.56	65.39
5	19.69	19.65	66.12	66.85
6	19.63	19.13	65.34	65.56
7	19.5	19.55	64.83	66.6
8	19.39	19.85	64.22	66.9
9	19.87	19.45	65.54	65.5
10	19.66	19.87	65.33	65.92
11	20.41	19.73	67.83	65.51
12	18.37	19.6	65.97	65.03
13	19.09	20.76	65.01	65.03
14	18.98	18.98	64.51	64.19
15	19.22	19.93	64.3	64.95
16	18.99	19.15	64.38	64.95
17	19.58	19.23	64.75	64.45
18	19.7	19.2	63.12	64.77
19	19	19.52	62.93	65.58
20	19.11	19.38	63.59	64.87
21	19.97	19.08	67.04	63.52
22	19.52	18.89	64.38	65.02
23	19.39	19.57	63.41	66.05
24	17.2	19.35	64.19	63.82
25	19.75	19.13	64.86	64.58
26	19.35	19.44	63.7	65.09
27	19.95	19.27	63.14	64.86
28	19.38	19.58	66	64.85
29	19.33	19.71	63.43	66.75
30	19	19.64	64.25	65.37
31	19.05	19.14	63.67	64.29
32	19.24	19.28	64.78	65.5
33	19.29	19.68	58.02	62.85
34	19.29	18.27	63.46	64
35	19.18	18.75	65.2	63.72
36	19.35	19.57	63.74	64.3

**Table 2:** The necessary computations for the proposed paired *t*-test.

Number of testing	*D*_*L*_	*D*_*U*_	*D*_*L*_^2^	*D*_*U*_^2^
1	-0.76	-1.01	0.3540	0.8567
2	-0.3	-1.25	0.0182	0.4978
3	-0.6	0.82	0.1892	1.1329
4	-1.38	-2.83	1.4762	11.3272
5	0.04	-0.73	0.0420	0.0238
6	0.5	-0.22	0.4422	1.2642
7	-0.05	-1.77	0.0132	0.9517
8	-0.46	-2.68	0.0870	5.2697
9	0.42	0.04	0.3422	1.7014
10	-0.21	-0.59	0.0020	0.0019
11	0.68	2.32	0.7140	14.7794
12	-1.23	0.94	1.1342	0.3073
13	-1.67	-0.02	2.2650	0.7150
14	0	0.32	0.0272	1.3558
15	-0.71	-0.65	0.2970	0.2658
16	-0.16	-0.57	2.5*E*-05	0.0130
17	0.35	0.3	0.2652	2.2332
18	0.5	-1.65	0.4422	0.0933
19	-0.52	-2.65	0.1260	5.4084
20	-0.27	-1.28	0.0110	0.4978
21	0.89	3.52	1.1130	27.6087
22	0.63	-0.64	0.6320	0.6962
23	-0.18	-2.64	0.0002	3.9029
24	-2.15	0.37	3.9402	0.8753
25	0.62	0.28	0.6162	3.0429
26	-0.09	-1.39	0.0056	0.4039
27	0.68	-1.72	0.7140	0.0382
28	-0.2	1.15	0.0012	3.2198
29	-0.38	-3.32	0.0462	8.1544
30	-0.64	-1.12	0.2256	0.8383
31	-0.09	-0.62	0.0056	0.0180
32	-0.04	-0.72	0.0156	0.0071
33	-0.39	-4.83	0.0506	19.1458
34	1.02	-0.54	1.4042	1.75403
35	0.43	1.48	0.3540	7.5867
36	-0.22	-0.56	0.0030	0.0041

## Data Availability

The data is given in the paper.
